# Remote Learning in Higher Education: Evidence from Poland

**DOI:** 10.3390/ijerph192114479

**Published:** 2022-11-04

**Authors:** Józef Ober, Anna Kochmańska

**Affiliations:** Department of Applied Social Sciences, Faculty of Organization and Management, Silesian University of Technology, Roosevelta 26-28, 41-800 Zabrze, Poland

**Keywords:** remote learning, quality of education, level of knowledge, higher education, COVID-19 pandemic

## Abstract

The COVID-19 pandemic has brought about a sudden transformation at universities. The previous mode of teaching has been replaced by remote education, the effectiveness of which depends, among other things, on the technological infrastructure of universities and the digital competence of lecturers and students. The main objective of this study is to evaluate remote learning in higher education from the students’ point of view. The uniqueness of the present research approach lies in the identification of four dimensions (socio-emotional, developmental, time-financial, and negative attitude) of students’ evaluation of remote learning in higher education. The survey was conducted on 999 students studying remotely, including 518 women and 481 men. Most of the students surveyed had been studying remotely for 1–2 years and were studying full-time for their first degree. The research tool consisted of 16 mixed survey questions. Six of them were related to sociodemographic factors (including those related to the respondents’ education), and eight were related to their experiences with and opinions about remote education, respectively. The remaining two questions were used to collect respondents’ evaluations of the degree of importance to them of various advantages and disadvantages of remote education. The research showed that among the advantages of remote learning for students, the most important are saving time, the possibility of studying at a university far from home (another city, another country), the possibility of combining work and study, and reduced commuting costs. On the other hand, the disadvantages of remote learning of greatest importance to students include the loss of social ties due to lack of contact with peers, feelings of fatigue resulting from excessive use of information and communication tools, and greater susceptibility to various forms of distraction. In addition, the shape of students’ education was relevant to the different dimensions of their evaluation of remote learning in higher education. The social-emotional size of remote learning is more important for students who study remotely in a blended mode (compared to uniform). The developmental dimension is essential for students who participate in remote learning activities for longer during the day. In addition, a more extended period of remote learning promotes the greater importance of the time-financial dimension when evaluating remote knowledge.

## 1. Introduction

Education has changed significantly since the beginning of the COVID-19 pandemic in 2020 [1], bearing in mind the fact that educational institutions around the world were closed for months [2]. During the pandemic, teachers changed their pedagogical practices and also developed new teaching sequences [3]. COVID-19 caused a massive transformation in many aspects of the educational landscape [4]. Within days, there was a shift from traditional teaching to online schooling [5] due to the danger of the virus being transmitted at a very rapid pace.

Universities had no choice but to develop an agile learning mechanism to connect different geographic locations, courses, and time zones [6]. Online learning thus became an indispensable element for sustaining higher education institutions [7]. It has begun to play a major role in supporting the educational process [8]. It is also worth noting that the transition from traditional “face-to-face” training to remote learning was a completely new experience for both students and teachers, to which both sides had to adapt [9]. Although many students were familiar with the Internet resources that complemented the knowledge acquired through traditional means, they did not expect a complete and rapid change in the mode of education [10]. The new form also posed a huge challenge for teachers, as they had to develop their digital skills in accordance with universal trends and their field of knowledge [11]. As a result, this led to high levels of stress in them [12]. Thus, in order to mitigate it and prepare teachers for teaching in the online environment, professional development strategies were designed, developed, and implemented [13].

In addition, many universities were not adequately equipped with the technical or organizational infrastructure to implement such a rapid and radical change [14]. However, the situation has improved significantly over time, thanks to the use of diverse information and communication technologies. Recently, these technologies have become increasingly sophisticated, especially in the new reality brought by COVID-19 [15].

In pedagogical terms, information and communication technologies (ICTs) are defined, on the one hand, as a set of technologies that contain, store, and disseminate information [16,17] (for example, e-books, videos or databases) and, on the other hand, as technologies that are designed for short-term communication (for example, social networks and smartphones) [18].

Online platforms such as Zoom, Microsoft Teams, Google Classroom, virtual learning environments, social media, and diverse group forums, among others are used for remote learning. They are also tested to overcome the limitations of virtual education. Lecturers must work together at the institutional level to improve them [19].

After analysis, it can be concluded that there are diverse definitions of remote education. It is an alternative to desktop learning, where teaching and learning takes place through platforms, using the Internet and computers or smartphones [20]. It can also be described as a powerful platform, in a new form for both students and researchers, thanks to the availability of advanced technological tools [21]. Thus, it can be stated that remote learning uses tools that provide more innovative and flexible learning experiences [22], with a greater focus on the recipient [23]. 

Remote delivery of information affects many areas of learning, so its effectiveness will affect the level of knowledge in ecology, environmental protection (shaping pro-environmental attitudes of its recipients) and health, among others [24]. It is also worth mentioning that such education contributes to economic growth, sustainable development, or gender equality [25].

Although remote education has been studied extensively over the past two decades, the detailed opinions of students on such activities during the COVID-19 pandemic seem very relevant due to their impact on the education system [26]. Nowadays, many universities have recognized the importance of e-learning as a core component of their teaching system. Accordingly, further research is being conducted to understand in more detail its advantages, disadvantages, and challenges in this type of educational institution [27]. The uniqueness of the present research approach lies in the identification of the dimensions (socio-emotional, developmental, time and financial, and negative attitudes) of students’ evaluation of remote learning in higher education. Therefore, referring to the above considerations, the main objective of the study presented in this paper is to evaluate remote learning in higher education from the students’ point of view. The following research questions were formulated:

- Question 1: What is the general opinion of students about remote learning compared to onsite learning?
- Question 2: What advantages of remote learning matter most to students?
- Question 3: What disadvantages of remote learning matter most to students?
- Question 4: In what dimensions do students evaluate remote learning in higher education?
- Question 5: Does the shape of students’ education matter to any of the dimensions of their evaluation of remote learning in higher education?
- Question 6: Does the importance of any of the dimensions of students’ evaluation of remote learning in higher education differ between male and female students?
- Question 7: What dimensions are particularly important in evaluations of remote learning in higher education for students with different forms of study and length of remote learning?
- Question 8: What dimensions are particularly important in evaluations of remote learning in higher education for students with different modes of study and length of remote learning?
- Question 9: What dimensions are particularly important in evaluations of remote learning in higher education for men and women with different lengths of remote learning?
- Question 10: What dimensions are particularly important in evaluations of remote learning in higher education for women and men studying in different modes?

The structure of the rest of the article begins with a review of the literature on the origins of remote education as well as popular tools used in this process. In addition, the advantages and disadvantages of distance education from the point of view of students are characterized along with suggestions for potential improvements in this area. This is followed by a description of the methodology used in this study and the results of the analysis and discussion. Finally, conclusions from a scientific and practical point of view are presented, as well as limitations and suggestions for further research.

## 2. Theoretical Background

In recent years, higher education institutions around the world have been experiencing rapid changes due to technological advances and social e-trends toward digitization [6]. Before the outbreak of the pandemic, universities in various countries represented varying levels when it came to the adoption of e-learning [28], even though remote learning is not an innovative educational approach. It has been integrated into higher education for many years [29]. The first educational programs based on correspondence and distance learning were initiated in the mid-19th century by the University of London. In 1873, the first official correspondence education program, known as the Society to Encourage Home Studies, was established in Boston, Massachusetts [30]. The year 1998 ushered in the growth of online programs, presented then by New York University. It is also worth mentioning here the University of Phoenix (known for conducting courses over the Internet), which began using online technology with CompuServe (an online service provider) in 1989, followed by the World Wide Web information system in 1991 [31].

As mentioned earlier, the pandemic reality forced universities to undertake efforts to make remote learning run smoothly, preventing obstacles from arising. Acting under exceptional circumstances, they have created virtual courses, using both synchronous and asynchronous forms depending on their content [32]. Although remote learning differs from traditional teaching [33], among other things, in terms of interactivity (whose deficit is caused by the absence of such important factors as social presence, social interaction and student satisfaction), it provides the opportunity for more people to continue learning [34]. What’s more, such a teaching model also shows outstanding commercial advantages [35].

Following on from earlier considerations, remote learning uses a wide range of tools that are tailored to the needs of both lecturers and learners. Moreover, many of them are free or licensed by the university [36]. These can include technology platforms [37] and applications that allow the users to create, edit, and share media files easily and quickly, without the need for storage on their device [38]. It is worth highlighting here interactive platforms such as Zoom, Skype, Google Meet or Microsoft Teams [39]. These are also referred to as video conferencing systems [40,41]. Their unquestionable advantage, in addition to the possibility of synchronous teaching, is the easy method for using them, which makes it possible to share differentiated teaching materials during the class, significantly enhancing the teaching process. In addition, numerous features allow, among other things, the creation of virtual classrooms where students can communicate with each other and perform varied tasks. They also participate in discussions monitored by the teacher [42].

WebVR technology has become an alternative to video conferencing platforms. It has made significant contribution to teaching and learning processes and provided new opportunities for interaction and collaboration through its tools. It is worth noting that it has been positively received by both listeners and lecturers [43]. 

One of the most valued aspects of remote education, is its flexibility in terms of time, which allows students to interact more with both lecturers and peers [11]. Asynchronous learning activities, where students learn at different times [44], on the other hand, allow for the ability to adjust the pace of learning to suit their needs. In addition, in virtual classrooms, students can access online resources and discuss with lecturers or group members anywhere [45]. Remote learning also contributes to students’ self-learning, self-education, and technological knowledge [46], increases the speed of knowledge acquisition and also develops the ability to learn and process information independently [47] through continuous learning of information and communication tools [48], and the development of digital competencies [49]. A significant advantage of remote education is also the saving of time and expenses [50] (related, among other things, to the cost of commuting) as well as the possibility of combining work with study.

In addition to the significant advantages of remote education outlined above, it also has drawbacks that affect its negative evaluation by participants in the classes. One of them is the fear of change that comes with learning new technologies. Interestingly, it can also occur in people who use computers [51]. In the group in question, it is also important to point out technical problems [52] that prevent active participation in activities. As a result of a significant reduction in social interaction [53], students may have a sense of isolation, which is further influenced by a change in the typical classroom environment [54]. Deprivation of contact with peers thus results in a loss of social ties.

Unfortunately, it also happens that many lecturers are insufficiently prepared to teach remotely [55] which affects the lack of adequate lecturer-student interaction [56]. Indeed, the behavior of teachers is an important factor that affects student engagement or lack thereof [57]. Attention should also be paid to the deterioration of students’ mental and physical health, symptoms of which include exhaustion and impaired concentration [58]. In addition, the pandemic reality promotes Internet addiction [59]. 

It is therefore worth considering how to improve the remote education process. One solution is to evaluate the tools that are used in such education. To do this, the tools that should be evaluated (in terms of their functionality) are identified. The next step is to conduct a survey questionnaire among students and also analyze the results [60].

Today’s lecturers are expected to take an active and collaborative approach to the learning process to develop students’ social learning experiences [61]. Accordingly, they need to invest continuously in their personal development to maximize their learning potential. They should also choose platforms that foster a friendly learning environment [62]. Therefore, it is recommended that universities develop training sessions or programs to motivate them [63]. The importance of feedback in the evaluation of remote education is also essential [64] because it improves the quality of education in the long term.

## 3. Materials and Methods

### 3.1. Research Tool

Empirical material was collected using the author’s survey questionnaire. The tool consisted of 16 mixed survey questions, six of them related to sociodemographic factors (including those related to the respondents’ education), and eight of them related to the respondents’ experiences with and opinions about remote learning, respectively. The remaining two questions were used to collect respondents’ evaluations of the degree of importance to them of various advantages and disadvantages of remote learning (respondents referred to individual items on a 5-point Likert scale). The reliability of the aforementioned questionnaire was tested by Cronbach’s coefficient of internal consistency alpha, the results of which indicated a satisfactory level of reliability (alpha = 0.64). The result obtained is in line with the recommendations for survey measurements [65].

The survey was conducted electronically via the Interankiety.pl online platform from 28 April 2022 to 16 June 2022. Students of higher education institutions in Poland were qualified for the survey. The survey was anonymous and voluntary. Completion of the survey was tantamount to the respondents’ consent to participate in the study. The sampling was random, 14,000 students were invited to complete the surveys, and the response rate was 7.12%.

### 3.2. The Object of Statistical Analysis

Evaluation of remote learning in higher education was the main focus of this analysis, and recoded ratings of the degree of importance of individual advantages and disadvantages of remote education on a point scale (1–5) were used as the main variables describing the above issue. Statistical analysis was aimed at finding out the areas of remote education to which the surveyed students paid special attention, as well as developing indicators to help verify evaluations (opinions) regarding remote learning in higher education.

Accordingly, a general analysis of the results of the survey was conducted, and an exploratory factor analysis was performed to identify the dimensions (indicators) of students’ evaluations of remote learning in higher education. Subsequently, the relationship of the developed dimensions of evaluation of remote learning in higher education to factors related to the education of the surveyed students was checked, i.e.:

- Remote learning mode,
- Average length (duration) of remote classes,
- Length of remote education, and
- Forms of study.

This was followed by a comparative analysis of men and women in terms of the importance of each dimension in evaluations of remote learning in higher education. Finally, an attempt was made to develop models for evaluating remote learning in higher education in conjunction with:

- The form of study and the length of remote learning,
- The mode of remote education and its length,
- Gender and length of remote education,
- Gender and form of study.

### 3.3. Methodology of Statistical Analysis

The collected research material was subjected to quantitative and descriptive analysis. The values of the analyzed measurable parameters were presented using the values of basic descriptive statistics, and the non-measurable ones were presented using counts (n) and percentages (%). The data analyzed in this way made it possible to verify the answer to research question 1. Assessment of the concordance of quantitative (measurable) variables was carried out using the Shapiro-Wilk test.

For statistical calculations, the following were used:

- Mann-Whitney U test to compare two groups in terms of quantitative or ordinal variables [66]. Glass’s rank biserial correlation coefficient was used as a measure of effect size [67]. The data analyzed in this way served to characterize the study sample and allowed to verify the answer to research questions 5 and 6.
- Spearman’s rank correlation to examine the interdependence between two quantitative and/or ordinal variables [66]. The data analyzed in this way made it possible to verify the answer to research question 5.
- Pearson’s χ^2^ independence test to assess the significance of the relationship between two variables of a nominal nature. As a measure of effect size, the Fi coefficient (φ) was used for 2 × 2 tables [66,67]. The data analyzed in this way served to characterize the study sample.
- Multidimensional cluster analysis was performed to extract homogeneous subsets of objects, more “similar” to objects from a given cluster compared to objects from other clusters. Clustering of objects was also performed in two ways: hierarchical agglomeration method and non-hierarchical k-means clustering. For the first method, Euclidean distance was used as the distance function. In the case of the second, Ward’s method was adopted [68]. The data analyzed in this way made it possible to verify the answer to research questions 2 and 3.
- Exploratory Factor Analysis (EFA) to detect common factors (of a new set of variables) responsible for the behavior of individual characteristics. Principal component method with Varimax crude rotation was used to extract factors [69]. The data analyzed in this way made it possible to verify the answer to research question 4.
- PROFIT analysis to assess the similarities of the studied objects in terms of selected characteristics and to develop a graphical presentation of the results of grouping objects and their relationship to the studied characteristics in the form of a perception map [70]. The data thus analyzed made it possible to verify the answer to research question 7–10.

The statistical package Statistica v.13.3 PL, Tulsa, OK, USA (using the “Marketing and Market Analysis” module for multivariate scaling and using PROFIT analysis) was used for the calculations. A 5% risk of inference error was assumed, *p* < 0.05 was considered a statistically significant level.

### 3.4. Characteristics of the Research Sample

The survey was conducted on a group of 999 remote learning students, including 518 women (51.85%) and 481 men (48.15%). The following tables present information on the age of the respondents and their educational situation (Table 1 and Table 2), with respect to both the total respondents and the groups distinguished by gender. They show that the vast majority of the subjects (82.98%) were aged 19–25; among women, the percentage of such cases was 83.01%, while among men it was 82.95% (there were no statistically significant differences between the two groups in this regard, as shown by analysis with the Mann-Whitney U test: Z = −0.14; *p* = 0.887; r_g_ = −0.01).

As for the educational situation, the majority of students surveyed had studied remotely for 1–2 years (53.35%), studied full-time (76.68%) for a 1st degree (78.78%) at a state university (84.08%). Women studied remotely slightly longer than men (Mrank_Women_ = 522.90 and Mrank_Men_ = 475.33); in the former group, there was a smaller proportion of those studying remotely for a period of less than a year (21.24% and 25.36%, respectively) and for 1–2 years (51.35% and 55.51%, respectively); more often, the period of such study lasted more than 2 years (19.13% and 27.41%, respectively). These differences reached statistical significance, as determined by the results of analysis with the Mann-Whitney U test: Z = 2.6; *p* < 0.01; r_g_ = 0.10. In addition, women were significantly more likely than men to study at a private university (19.11% and 12.47%, respectively), as demonstrated by analysis with Pearson’s χ^2^ test: χ^2^(1) = 8.21; *p* < 0.01; φ = −0.09. There were no significant differences between the two groups in terms of the form of study: χ^2^(1) = 2.33; *p* = 0.127; φ = −0.05 and level of study: χ^2^(1) = 0.58; *p* = 0.446; φ = 0.02.

The sample size formula for qualitative characteristics was used to estimate the minimum sample size [71]. When calculating the minimum sample size, data from the Central Statistical Office (CSO) on 1,215,300 university students in Poland in the 2020/2021 academic year were taken into account [72]. In addition, a 95% probability was assumed that the result obtained in the study would not deviate from the actual value in the population by more than 5%. According to these estimates, the minimum sample size is 384 subjects. Thus, the achieved sample size (N = 999) exceeded its minimum level by nearly three times. 

## 4. Results and Discussion

### 4.1. Analysis of Total Results

#### 4.1.1. Advantages of Remote Learning in Higher Education

The analysis in this section will answer the second research question: What advantages of remote learning matter most to students?

The surveyed students primarily perceived as advantages of remote education the reduction in commuting costs (81.58% of responses “They are an advantage to the maximum degree”), the possibility of studying at a university far from home (75.28% respectively), saving time (64.06%), and the possibility of combining professional work with studying (56.86%). Opinions were more divided on the other advantages of studying remotely, with generally a minimum of moderate importance of these factors to respondents (Table 3).

In order to compare the ratings of the importance more accurately to the surveyed students of the individual advantages of remote learning, their responses were recorded on a point scale from 1 to 5, where 1 point was awarded for the answer “They are not an advantage,” and 5 points were awarded for the answer “They are a maximum advantage” (thus, a higher number of points indicated the greater importance of a particular advantage). Further analysis of these results confirmed that the most important advantages of remote learning for respondents were the reduction in commuting costs (M = 4.69; SD = 0.76) and the possibility of studying at a university far from home (M = 4.56; SD = 0.9). Slightly less important to the students surveyed were the advantages of saving time (M = 4.4; SD = 0.97), the opportunity to combine work and study (M = 4.2; SD = 1.13) and flexible study time (M = 4.03; SD = 1.11). Next in this respect were the development of digital competencies (M = 3.7; SD = 1.23), the opportunity to adjust the pace of learning to one’s needs (M = 3.67; SD = 1.29) and the opportunity to learn about information and communication tools (M = 3.51; SD = 1.25). On the other hand, the least important for the respondents was the quick acquisition of knowledge (M = 3.23; SD = 1.25) (Table 4).

**Figure 1:**
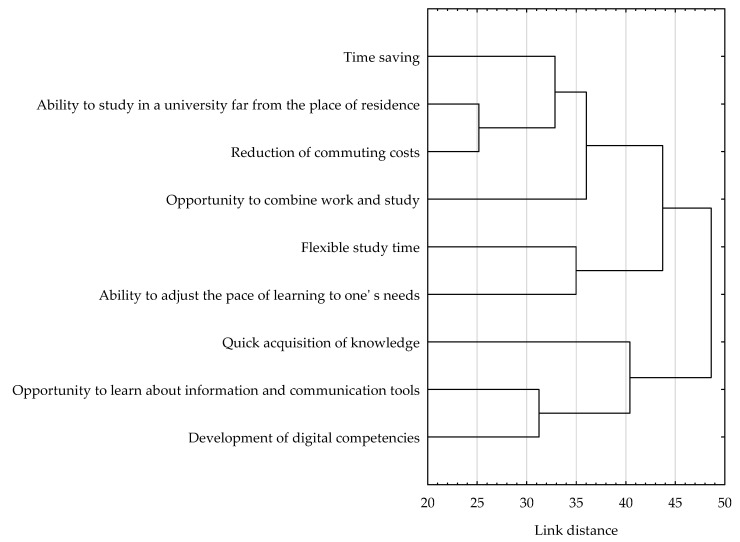
Dendrogram obtained for the analyzed advantages of remote learning (results of cluster analysis using agglomeration).

A multivariate cluster analysis was further used to identify groups of advantages of remote education of similar significance to the respondents. The results of this analysis, using the agglomerative method, showed that when evaluating remote learning, students paid similar attention to issues related to time savings, the ability to study at a university far from home, reduced commuting costs, and the opportunity to combine work and study. The second identified group of advantages of similar importance were issues related to flexible study time and the ability to adjust the pace of learning to one’s needs. In addition, similarities were found in the assessment of the importance of the advantages of remote education concerning the quick acquisition of knowledge, the opportunity to learn about information and communication tools and the development of digital competencies (Figure 1).

The results of the cluster analysis using the non-hierarchical feature clustering method, so-called k-means clustering, fully matched the results of this analysis using the agglomerative method. The individual advantages of remote learning were divided into three analogous clusters, consisting of the same advantages in terms of significance for the students surveyed. Further analysis of descriptive statistics for the advantages included in each cluster showed that the advantages included in cluster 3 (i.e., time savings, the ability to study at a university far from home, the opportunity to combine work and study, and reduced commuting costs) were of the highest importance (M = 4.46; SD = 0.97). The advantages included in the other two clusters were clearly of lesser importance (M_Cluster no. 1_ = 3.85; SD_Cluster no. 1_ = 1.22 and M_Cluster no. 2_ = 3.48; SD_Cluster no. 2_ = 1.26) (Table 5).

The advantages of remote learning outlined are consistent with the opinions of students from two general English courses at a university located in Abu Dhabi in the United Arab Emirates. According to them, the most frequently mentioned positive aspects of online learning were cost and time efficiency, safety, convenience, and increased opportunity to participate in class [73]. On the other hand, undergraduate students participating in one of the courses provided by a large university located in the Midwestern part of the United States noted, among other things, the flexibility and ability to revisit materials, the acquisition of new computer skills, and the support of lecturers, which enabled them to better adapt to remote learning and also allowed them to complete the courses [74]. Referring to a survey conducted among students of the information technology department at Benghazi University, it can be concluded that e-learning is helpful, and one of the most significant applications is the mapping of the learned scientific method through the electronic (multimedia) form. In addition, Students agree that e-learning is functional, helps them stay safe, and improves their academic level [27]. It is also worth noting the research in this field conducted at the University of Lahore. Students emphasize that this instruction contributed to their focus and increased self-reliance [75].

#### 4.1.2. Disadvantages of Remote Learning in Higher Education

The analysis in this section will answer the third research question: What disadvantages of remote learning matter most to students?

In assessments of the degree of significance of most advantages of remote education, the students surveyed were divided. For every second person surveyed, disadvantages such as fear of change (50.85% of responses “They are not a disadvantage”) and the possibility of Internet addiction (51.65% respectively) were not important. For other disadvantages, respondents’ opinions were divided, and in general the highest percentage was for moderate ratings (“They are a moderate disadvantage”). The exceptions were the feeling of isolation and/or alienation, where the largest proportion indicated that the aforementioned disadvantage is not important (29.13%), and the disappearance of social ties due to lack of contact with peers and the feeling of fatigue resulting from excessive use of information and communication tools, where the largest proportion were those indicating the maximum degree of importance (respectively: 23.32% and 22.42% of “They are a maximum disadvantage” responses) (Table 6).

Analogous to the advantages of remote education, in order to more accurately compare the ratings of the importance for the surveyed students of the individual disadvantages of remote education, their responses were recorded on a point scale from 1 to 5, where 1 point was awarded for the answer “They are not a disadvantage”, while 5 points were awarded for the answer “They are a maximum disadvantage” (the higher number of points indicated the greater importance of a given disadvantage). An analysis of the descriptive statistics values of the scoring of individual disadvantages of remote education showed that the greatest importance for the students surveyed was the loss of social ties due to lack of contact with peers (M = 3.16; SD = 1.43), greater susceptibility to various forms of distraction (M = 3.1; SD = 1.41), and feelings of fatigue due to excessive use of information and communication tools (M = 3.04; SD = 1.45). Slightly less important were technical problems during remote classes (M = 2.97; SD = 1.15), lack of sufficient lecturer-student interaction (M = 2.85; SD = 1.28), feelings of isolation and/or alienation (M = 2.72; SD = 1.45), and insufficient preparation of lecturers for remote classes (M = 2.6; SD = 1.28). In contrast, students attributed the least importance in their evaluation of remote learning to the possibility of Internet addiction (M = 1.97; SD = 1.24) and fear of change (M = 1.95; SD = 1.19). At the same time, it should be borne in mind that the average ratings of the importance of individual disadvantages of remote education were at the maximum moderate level, and the values of standard deviations testify to the relatively large variation of the surveyed group of students in the aforementioned ratings (Table 7).

**Figure 2:**
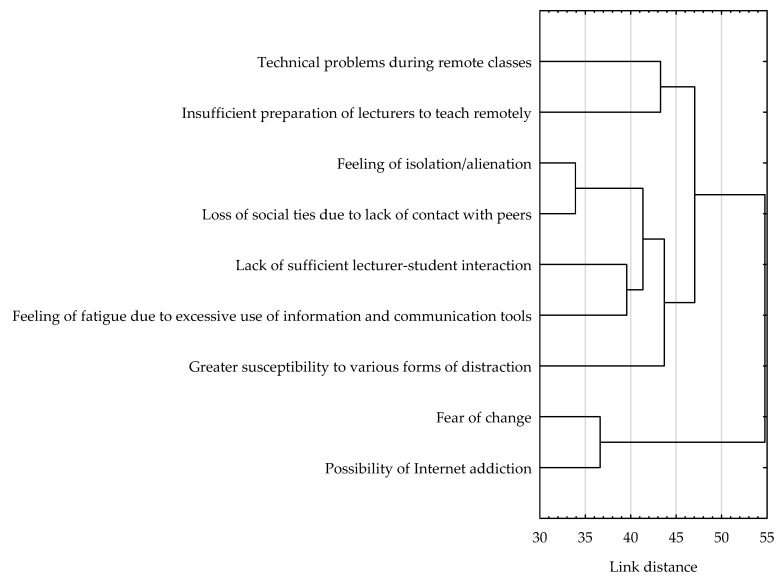
Dendrogram obtained for the analyzed disadvantages of remote learning (results of cluster analysis using agglomeration).

Based on the results of the cluster analysis using the agglomerative method, it was found that technical problems and insufficient preparation of lecturers for remote instruction were of similar importance to the surveyed students in their evaluation of remote education. Another cluster of disadvantages of remote education of similar significance to the students was the feeling of isolation or alienation, the loss of social ties due to the lack of contact with peers, the lack of sufficient lecturer-student interaction, the feeling of fatigue due to excessive use of information and communication tools, and greater susceptibility to various forms of distraction. The third and final cluster combined the disadvantages of fear of change and the possibility of Internet addiction (Figure 2).

The cluster analysis conducted using k-means clustering confirmed the groups of disadvantages of remote education identified by the previous method in the opinion of the surveyed students. As can be read from the distribution of descriptive statistics on the individual groups of advantages, the issues of greatest importance to the surveyed students were those related to the limitations of interacting with others and the course of remote learning (including a sense of isolation and/or alienation and greater susceptibility to various forms of distraction) (M = 2.97; SD = 1.41). Slightly less importance was given to technical issues of remote learning (i.e., technical problems during remote classes and insufficient preparation of lecturers for remote classes) (M = 2.78; SD = 1.23). Disadvantages touching on fears or threats associated with the remote mode of learning (i.e., fear of change and the possibility of Internet addiction) were of least importance to respondents (M = 1.96; SD = 1.21) (Table 8).

Similar to Polish students, students from two general English courses at a university in Abu Dhabi also cited distraction and reduced concentration as the disadvantages of remote learning [73]. When analyzing the negatives of remote learning, it is also worth noting a study conducted among undergraduate students majoring in administration at a Swiss business school. They pointed out how burdensome full-time remote education is. Students became demotivated in the so-called online environment due, among other things, to the lack of personalized information [76]. Referring to the survey above of students in the department of information technology at Benghazi University, it can be said that the main barriers to remote education are the difficulty associated with its introduction and the low quality of online services that prevent its practical use. Respondents show that there are limitations to e-learning and that its most significant downside is that it reduces the workload of teaching staff and increases the pressure on students [27]. It is also worth referring to a study conducted among 2795 German students. They proved that students, as a result of the sudden transition to remote learning mode, observed an increased stress level [77]. This is confirmed by a study conducted among respondents at a university in northern Malaysia who admitted that anxiety and depression symptoms were exceptionally high during the ongoing COVID-19 pandemic and online learning. Indeed, both assignments and exams conducted remotely were a significant source of stress for students [78].

#### 4.1.3. Other Factors Regarding Remote Learning in Higher Education

The analysis in this section will answer the first research question: What is the general opinion of students about remote learning compared to onsite learning?

More than half of the surveyed students (51.75%) did their remote learning in mixed mode (synchronous and asynchronous), with a significant percentage of the remainder learning in synchronous mode (40.04%). At the same time, the majority of respondents (59.06%) considered mixed mode to be the best for transferring knowledge. In terms of the average length of remote classes, the largest number of respondents, or almost every second person (48.65%), took classes for 3–5 h a day, and a slightly smaller share were those who studied remotely for an average of 6–8 h a day (39.74%).

Given a choice of the form of study, surveyed students mostly indicated remote learning (25.53% of responses “Definitely remote learning” and 27.03% “Rather remote learning”, respectively). What’s more, remote learning was generally considered by respondents to be as effective as on-site learning (20.02% of “Definitely yes” responses and 31.93% of “Definitely no” responses).

During the remote learning conducted at the university of the surveyed students, the majority of them (64.16%) were provided with the opportunity to express their opinion on this form of education. On the other hand, when asked to evaluate the level of remote learning at their university, the respondents expressed mostly positive opinions, i.e., almost half of the respondents (49.15%) evaluated the above form rather well, and one in five respondents (23.82%)-definitely well. On the other hand, as actions that could improve remote learning in the future, the surveyed students indicated in the vast majority (74.87%) the organization of training for lecturers on the varied possibilities of using remote education tools. 

### 4.2. Examination of Selected Relationships on Evaluations of Remote Learning in Higher Education

#### 4.2.1. Factor Model for Evaluating Remote Learning in Higher Education

The analysis in this section will answer the fourth research question: In what dimensions do students evaluate remote learning in higher education? 

An exploratory factor analysis (EFA) using the principal components method with Varimax rotation was used to estimate indicators (subscales) for evaluating remote education in higher education system. Point scale ratings of individual advantages and disadvantages of remote education were used as variables in this analysis, i.e.:

- Item 1 Saving time
- Item 2-Flexible study time
- Item 3-Ability to adjust the pace of learning to one’s needs
- Item 4-Opportunity to study in a university far from place of residence (another city, another country)
- Item 5-Quick acquisition of knowledge
- Item 6-Opportunity to learn about information and communication tools
- Item 7-Opportunity to combine professional work with study
- Item 8-Reduction in commuting costs
- Item 9-Development of digital competencies
- Item 10-Technical problems during remote classes
- Item 11-Feeling of isolation/alienation
- Item 12-Loss of social ties due to lack of contact with peers
- Item 13-Lack of sufficient lecturer-student interaction
- Item 14-Feeling of fatigue due to excessive use of information and communication tools
- Item 15-Insufficient preparation of lecturers to teach remotely
- Item 16-Fear of change
- Item 17-Possibility of Internet addiction
- Item 18-Greater susceptibility to various forms of distraction.

Going ahead with the aforementioned analysis, all the necessary assumptions were checked. Accordingly, correlations between items (variables) were examined. The results of these tests are presented below (Table 9 and Table 10). As can be read from them, all variables were statistically significantly correlated with each other. Therefore, there was no need to remove any of the items from further analysis.

**Figure 3:**
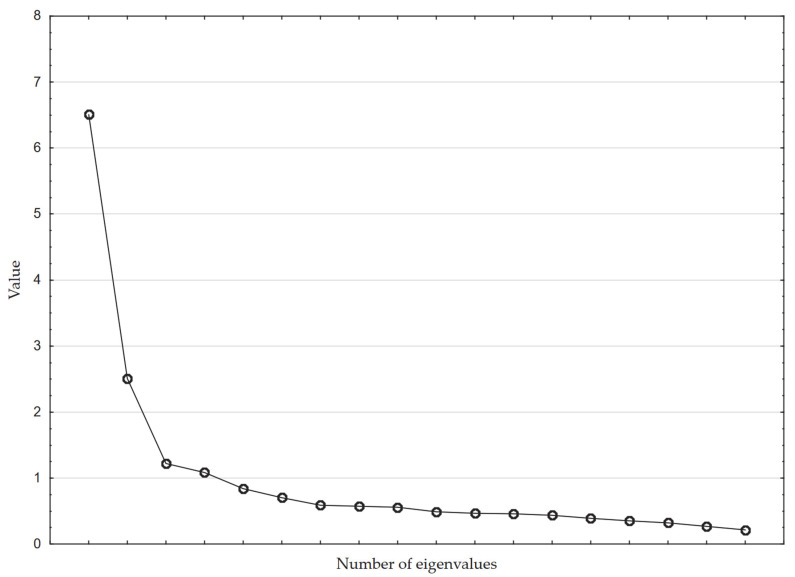
Scree plot (Cattell criterion).

The validity of the factor analysis was also checked. The results were fully satisfactory and confirmed the validity of conducting the factor analysis (KMO = 0.899; Bartlett’s test: χ^2^ = 8091.56; df = 153; *p* < 0.001). The principal component method was used to determine the number of factors to be extracted, and the Kaiser-Gutman criterion and the scree plot were examined. According to the first criterion, factors with eigenvalues less than one should be eliminated, while in the standard of the scree plot (the Cattell criterion), representing the descending sorted eigenvalues of factors, the place of flattening of the curve should be determined, which separates significant factors from those considered insignificant (factors with low eigenvalues in the flat part of the plot, the so-called scree plot, should be interpreted as factors with information noise significance) [79]. It turned out that the first criterion indicated the adoption of a solution consisting of four factors (Table 11), while the second criterion indicated the adoption of three factors, respectively (Figure 3). Therefore, trials were undertaken for both solutions, and it was checked which one leads to the best interpretable arrangement, which is in accordance with recommendations [66]. On this basis, a four-factor solution was decided upon.

The classification of a particular item into a given factor was based on an analysis of the factor loadings. As a criterion for the inclusion of an item in a given factor, a loading value greater than 0.50 was adopted, at the same time with a low degree of saturation with the remaining factor. This left 16 items comprising four indicators that determine the evaluation of remote learning in higher education. The extracted factors explain a total of 62.99% of the variance in the results. Table 12 shows the results of the factor analysis conducted, the percentage of explained variance and the reliability of each indicator.

Subsequently, the items included in the individual factors were analyzed in order to precisely define the extracted indicators for evaluating remote learning in higher education. It was assumed that the aforementioned evaluation included the following dimensions (subscales):

- The socio-emotional dimension (feeling of isolation or alienation, loss of social ties due to lack of contact with peers, lack of sufficient lecturer-student interaction, feeling of fatigue due to excessive use of information and communication tools, and greater susceptibility to various forms of distraction), having a negative character due to the fact that the items included in it represent exclusively disadvantages of remote learning.
- The developmental dimension (quick acquisition of knowledge, the opportunity to learn about information and communication tools, and the development of digital competencies), which has a positive character due to the fact that the items included in it are exclusively advantages of remote education.
- The time and financial dimension (saving time, flexible study time, the opportunity to study at a university far home, the opportunity of combining work and study, and the reduction in commuting costs) of a positive nature, as well.
- The dimension of negative attitudes (insufficient preparation of lecturers to teach remotely, fear of change and the possibility of Internet addiction) of a negative nature, as is clear from the name given to it.

Finally, descriptive statistics were calculated for the created dimensions of evaluation of remote learning in higher education, based on the average ratings of the importance of the individual advantages and disadvantages included in the aforementioned dimensions. The results showed that the surveyed students, when evaluating remote learning, focused mainly on its positive sides, especially the time and financial dimension (M = 4.38; SD = 0.72). The lowest results were recorded for the dimension of negative significance (M = 2.17; SD = 0.97), which means that the surveyed students, when evaluating remote learning, attributed the least importance to the potential risks associated with this form of learning (Table 13).

#### 4.2.2. The Influence of Selected Education-Related Factors on the Evaluation of Remote Learning in Higher Education in Its Various Dimensions

The analysis in this section will answer the fifth research question: Does the shape of students’ education matter to any of the dimensions of their evaluation of remote learning in higher education?

***Mode of remote learning vs. evaluation of this form of learning in higher education***

The socio-emotional dimension as the only indicator of evaluation of remote learning in higher education was differentiated by the mode of this form of learning of the respondents. It turned out that students learning remotely in a uniform mode paid less attention to the above-mentioned dimension of this form of education (M_Uniform_ = 2.89; SD_Uniform_ = 1.12) compared to respondents educated in mixed mode (M_Mixed_ = 3.05; SD_Mixed_ = 1.16). This difference reached statistical significance, as shown by analysis with the Mann-Whitney U test: Z = −2.23; *p* < 0.05; r_g_ = −0.08.

The other dimensions of evaluation of remote learning in higher education did not depend on the mode of this learning among the students surveyed. Those studying in a uniform mode paid slightly more attention to the developmental dimension than those studying in a mixed mode (M_Uniform_ = 3.54; SD_Uniform_ = 1.07 and M_Mixed_ = 3.43; SD_Mixed_ = 1.04), while the time and financial dimension was less important for the former group (M_Uniform_ = 4.35; SD_Uniform_ = 0.75 and M_Mixed_ = 4.4; SD_Mixed_ = 0.68) as was the dimension of negative attitudes (M_Uniform_ = 2.13; SD_Uniform_ = 0.96 and M_Mixed_ = 2.21; SD_Mixed_ = 0.97). However, the differences noted were found to be statistically insignificant, as determined by the results of analysis with the Mann-Whitney U test, both for the development dimension: Z = 1.88; *p* < 0.06; r_g_ = 0.07; time and financial dimension: Z = −0.42; *p* = 0.676; r_g_ = −0.02; as well as the dimension of negative attitudes: Z = −1.4; *p* = 0.16; r_g_ = −0.05.

***Average length of remote classes vs. evaluation of remote learning in higher education***

Analysis by Spearman’s rank correlation method showed that there was a positive and statistically significant relationship between the average length of remote classes for the students surveyed and their evaluation of remote learning in the development dimension: R = 0.08; t(N−2) = 2.57; *p* < 0.05. This means that the longer time allocated by the respondents to participate in remote classes was conducive to a better evaluation of this form of learning in the aforementioned dimension. The longer the respondents’ remote classes lasted, the more importance they attributed to the development dimension when evaluating this form of learning (from M_3–5 h_ = 3.42; SD_3–5 h_ = 1.04 to M_>8 h_ = 3.74; SD_>8 h_ = 1).

The average length of the respondents’ remote classes, on the other hand, was not significant for their evaluation of remote learning in the other dimensions. In the different groups distinguished by the average daily time of remote classes, while evaluating the above education attention was paid to its socio-emotional dimension at the level from M_> 8 h_ = 2.88; SD_> 8 h_ = 1.16 to M_6–8 h_ = 2.99; SD_6–8 h_ = 1.15, time and financial-respectively: from M_6–8 h_ = 4.33; SD_6–8 h_ = 0.76 to M_>8 h_ = 4.42; SD_>8 h_ = 0.68. while the dimension of negative attitudes mattered to respondents at levels ranging from M_3–5 h_ = 2.17; SD_3–5 h_ = 0.95 to M_> 8 h_ = 2.2; SD_> 8 h_ = 0.99. Based on the results of Spearman’s rank correlation analysis, it was found that there was no statistically significant relationship between the average length of the respondents’ remote classes and their evaluation of remote learning in higher education in both the socio- emotional dimension: R = –0.01; t(N–2) = −0.41; *p* = 0.678; time and financial: R = −0.01; t(N–2) = −0.29; *p* = 0.773; as well as in the dimension of negative attitude: R = 0; t(N–2) = 0.1; *p* = 0.92.

***Length of remote learning vs. evaluation of this form of learning in higher education***

Based on the results of analysis by Spearman’s rank correlation method, a positive and statistically significant relationship between the length of remote education and the evaluation of this form of education in higher education in the development dimension: R = 0.08; t(N-2) = 2.61; *p* < 0.01 and the time and financial dimension: R = 0.12; t(N-2) = 3.91; *p* < 0.001 were observed. The direction of these correlations indicates that the longer period of remote learning of the respondents was associated with their greater perception of the positive dimensions of this form of education. The longer the students surveyed studied remotely, the more important it was to assess this form of learning, be it the development dimension (from M_≤1 year_ = 3.3; SD_≤1 year_ = 1.1 to M_>2 years_ = 3.56; SD_>2 years_ = 1.07) or the time and financial dimension, for them (from M_≤1 year_ = 4.22; SD_≤1 year_ = 0.82 to M_>2 years_= 4.48; SD_>2 years_ = 0.65).

The socio-emotional dimension and the dimension of negative attitudes toward remote learning in evaluations of this form of education in higher education were not significantly related to the length of remote learning of the students surveyed. Admittedly, the longer the respondents studied remotely, the less important the socio-emotional dimension was for them in the above-mentioned evaluations (from M_≤1 year_= 3.04; SD_≤1 year_ = 1.13 to M_>2 years_ = 2.85; SD_>2 years_ = 1.15). On the other hand, the importance of the negative attitude dimension was similar in the groups distinguished by the length of remote learning and oscillated between M_>2 years_= 2.12; SD_>2 years_ = 1 a M_1–2 years_ = 2.2; SD_1–2 years_ = 0.94. As the analysis by Spearman’s rank correlation method showed, there was no statistically significant relationship between the length of remote education for the respondents and their evaluation of this form of learning in higher education in both the socio- emotional dimension: R = -0.05; t(N-2) = −1.7; *p* < 0.09; and negative attitude dimension: R = −0.02; t(N-2) = −0.64; *p* = 0.524.

***Form of study vs. evaluation of remote learning in higher education***

The evaluation of remote learning in higher education in each dimension was significantly differentiated by the form of study of the students surveyed. It turned out that full-time students-when evaluating remote learning-paid more attention than part-time students to the socio- emotional dimension of this form of education (M_Full-time_ = 3.03; SD_Full-time_ = 1.15 and M_Part-time_ = 2.77; SD_Part-time_ = 1.12) and the dimension of negative attitudes, that is, the potential threats posed by it (M_Full-time_ = 2.2; SD_Fulltime_ = 0.95 and M_Part-time_ = 2.09; SD_Part-time_ = 1.01). In contrast, part-time students were more likely than full-time students to focus their evaluations on the development dimension of remote learning (M_Part-time_ = 3.8; SD_Part-time_ = 1.03 and M_Full-time_ = 3.38; SD_Full-time_ = 1.04) and time and financial dimension (M_Part-time_ = 4.48; SD_Part-time_= 0.74 and M_Full-time_ = 4.34; SD_Full-time_ = 0.71). Thus, the negative sides of remote learning were more often perceived by full-time students, while the positive sides were perceived by part-time students, respectively. The differences found reached statistical significance, as shown by analysis with the Mann-Whitney U test. This was true for both the socio-emotional dimension: Z = 3.08; *p* < 0.01; r_g_ = 0.13; the development dimension: Z = −5.44; *p* < 0.001; r_g_ = −0.24; the time and financial dimension: Z = −3.72; *p* < 0.001; r_g_ = −0.16; and the negative attitude dimension: Z = 2.08; *p* < 0.05; r_g_ = 0.09.

#### 4.2.3. Comparative Analysis of Men and Women in Terms of Evaluation of Remote Learning in Higher Education in Its Various Dimensions

The analysis in this section will answer the sixth research question: Does the importance of any of the dimensions of students’ evaluation of remote learning in higher education differ between male and female students?

The gender of the students surveyed was significant in their evaluation of remote learning in higher education along two dimensions. When evaluating the above-mentioned form of education, women attributed greater importance than men to the development dimension (M_Women_ = 3.65; SD_Women_ = 1.02 and M_Men_ = 3.3; SD_Men_ = 1.07) and time and financial dimension (M_Women_ = 4.49; SD_Women_ = 0.65 and M_Men_ = 4.26; SD_Men_ = 0.77). Based on the results of the analysis with the Mann-Whitney U test, the above differences were found to be statistically significant, both in terms of grades in the development dimension: Z = 5.11; *p* < 0.001; r_g_ = 0.19; and in time and financial dimension: Z = 5.27; *p* < 0.001; r_g_ = 0.19.

For the other two dimensions of evaluation of distance learning in higher education, there were no significant differences between the women and men surveyed. Although, as in the case of the previous dimensions, women paid more attention in their evaluations than men to both the socio-emotional dimension (M_Women_ = 3.03; SD_Women_ = 1.18 and M_Men_ = 2.91; SD_Men_ = 1.11), as well as the dimension of negative attitude (M_Women_ = 2.25; SD_Women_ = 1.03 and M_Men_ = 2.1; SD_Men_ = 0.89). However, these differences did not reach statistical significance, as shown by analysis with the Mann-Whitney U test for both dimensions, i.e., socio-emotional: Z = 1.6; *p* = 0.11; r_g_ = 0.06 and negative attitude: Z = 1.69; *p* < 0.09; r_g_ = 0.06 (Table 14).

#### 4.2.4. Models of Evaluation of Remote Learning in Higher Education in Its Various Dimensions in Connection with Selected Factors

The final step in the statistical analysis was an attempt to build models for evaluating remote learning in higher education across different groups using PROFIT analysis (PROperty FITting). The purpose of this was to find out the dimensions of the aforementioned evaluation that are particularly relevant to groups of students distinguished by form of study, length of remote education, mode of remote education, or gender.

***Evaluation of Remote Learning in Higher Education in Its Various Dimensions in Relation to the Form of Study and the Length of Remote Learning***

The analysis in this section will answer the seventh research question: What dimensions are particularly important in evaluations of remote learning in higher education for students with different forms of study and length of remote learning?

The first model developed concerned the evaluation of remote learning in higher education in groups distinguished by form of study and length of remote learning. The analyzed objects in this model were groups of students:

- Studying full-time and studying remotely for up to 1 year [S(<1)],
- Studying full-time and studying remotely for 1–2 years [S(1–2)],
- Studying full-time and studying remotely for more than 2 years [S(>2)],
- Studying part-time and studying remotely for up to 1 year [N(<1)],
- Studying part-time and studying remotely for 1–2 years [N(1–2)],
- Studying part-time and studying remotely for more than 2 years [N(>2)].

On the other hand, the variables (characteristics) were individual dimensions of evaluation of remote learning in higher education, such as:

- Socio-emotional dimension [W1],
- Development dimension [W2],
- Time and financial dimension [W3],
- Negative attitude dimension [W4].

To develop the model, the average scores of each dimension of evaluation of remote learning in higher education recorded in the above-mentioned groups were used. Detailed data in this regard are presented in Table 15.

First, multidimensional scaling was carried out to develop a graphic presentation of the structure of similarity (or dissimilarity) between the analyzed objects in relation to a selected set of variables (characteristics). The identical nature of the analyzed characteristics (5-point Likert scale) as variables precluded the need to standardize them. In the course of multidimensional scaling, the classical Euclidean distance was used and, consequently, the four characteristics describing the six objects under study were reduced to two dimensions. The STRESS coefficient for multidimensional scaling taking into account all characteristics was 0.00, which meant high reliability of the results of the multidimensional scaling procedure. The resulting multidimensional scaling map shows that those who study full-time and study remotely for a minimum of one year and those who study part-time and study remotely for a period not exceeding one year are similar in terms of their evaluation of remote learning in its various dimensions. The remaining groups deviate from all of them in this regard (they are not close to any).

The next step was to verify the fit of individual objects. Accordingly, the results of the regression analysis, in which the dependent variable was the individual dimensions of evaluation of remote learning in higher education, and the explanatory variables were the values of the two dimensions for each unit, obtained by multidimensional scaling, were evaluated: Dim. 1 and Dim. 2. The above analysis showed that all the evaluation dimensions studied had a very high impact on the differentiation of the units studied. The lowest fit occurred for the socio-emotional dimension (R^2^ = 0.86) and the highest for the development dimension, respectively (R^2^ = 0.99) (Table 16). This form of results indicated that there was no need to limit the number of characteristics studied in the model.

**Figure 4:**
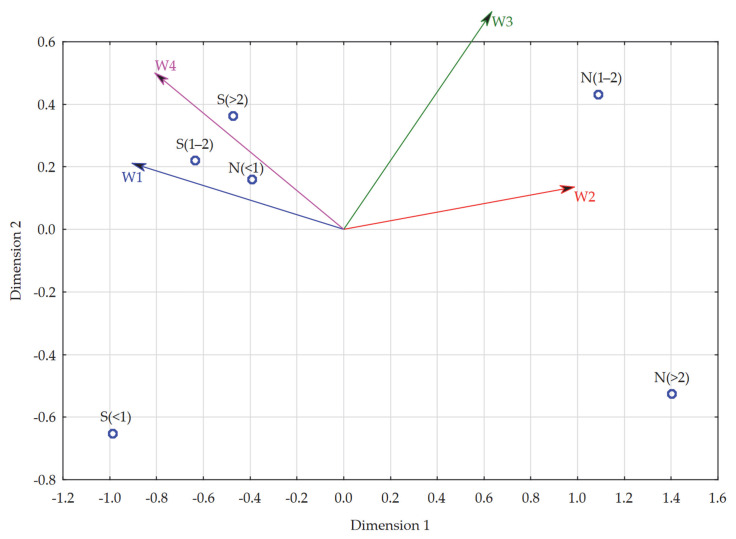
Biplot including the result of multidimensional scaling for individual objects (groups distinguished by the form of study and length of remote learning) based on each dimension of evaluation of remote learning in higher education.

A PROFIT analysis was conducted at the end of the effort to develop a model for evaluating remote learning in higher education in each of its dimensions in relation to the form of study and length of remote learning, and the result is shown in the chart below (Figure 4). As can be read from it, the socio-emotional dimension (W1) was particularly important for full-time students and remote learners for a period of 1–2 years, and part-time students and remote learners for a period not exceeding one year. In addition, full-time students with a remote learning period of more than 2 years also paid relatively high attention to the above dimension. All three of the aforementioned groups focused to a similarly large extent in their evaluations of remote learning on its negative attitude dimension (W4), and thus drew attention to the potential dangers of remote learning. This means that the above groups focused to a relatively large extent on the negative sides of remote learning in their evaluations. Among the other groups, part-time students who studied remotely for a period of 1–2 years paid attention to the positive sides of remote learning in their evaluations, i.e., they focused to a similar extent on the development (W3) and time and financial (W3) dimensions. For part-time students, for whom the period of remote learning was more than 2 years, none of the dimensions of evaluation of remote learning were of particular importance, with the development dimension (W2) being the closest to them. It should additionally be noted that the latter group was definitely not guided by dimensions with negative connotation in their evaluations (W1 and W4). The situation was similar for full-time students, for whom the aforementioned period did not exceed a year, where also none of the dimensions of evaluation of remote learning were of particular importance; the socio-emotional dimension was closest to them (W1), while they were definitely far from positive issues regarding remote learning (W2 and W3).

***Evaluation of Remote Learning in Higher Education in Its Various Dimensions in Connection with the Mode of Remote Learning and Its Length***

The analysis in this section will answer the eighth research question: What dimensions are particularly important in evaluations of remote learning in higher education for students with different modes of study and length of remote learning?

The second model developed concerned the evaluation of remote learning in higher education in groups distinguished by mode and length of remote learning. The methodology for the development of this model was analogous to the previous one, so the information on the methods used is not cited again below.

In the construction of this model, the objects analyzed were groups of students:

- Studying remotely in a uniform mode for up to 1 year [J(<1)],
- Studying remotely in a uniform mode for a period of 1–2 years [J(1–2)],
- Studying remotely in a uniform mode for more than 2 years [J(>2)],
- Studying remotely in a mixed mode for up to 1 year [J(<1)],
- Studying remotely in a mixed mode for a period of 1–2 years [J(1–2)],
- Studying remotely in a mixed mode for more than 2 years [J(>2)].

On the other hand, the variables (characteristics) were, as in the case of the previous model, the dimensions of evaluation of remote learning in higher education. As before, the average scores of each dimension of evaluation of remote learning in higher education recorded in the above-mentioned groups were used to develop the model. The aforementioned results are detailed below (Table 17).

The STRESS coefficient for multidimensional scaling considering all characteristics was 0.00, indicating the high reliability of the results of the multidimensional scaling procedure. From the resulting map, it can be read that those who have been studying remotely in mixed mode for a minimum of one year are similar in terms of their evaluation of remote education in its various dimensions. Analogous similarities were observed for those studying remotely for up to a year regardless of the mode. Students studying remotely in a uniform mode for a minimum of one year differed in their evaluations from the other groups (including relatively from each other).

The results of the regression analysis conducted for each dimension of evaluation of remote learning in higher education (dependent variables) and the values of the two dimensions for each unit obtained by multidimensional scaling: Dim. 1 and Dim. 2 (explanatory variables) indicate a very good fit between the individual objects. All of the evaluation dimensions studied had a high impact on the differentiation of the surveyed units. The lowest fit was for the dimension of negative attitude (R^2^ = 0.73), and the highest for the time and financial dimension, respectively (R^2^ = 0.96) (Table 18). Thus, there was no need to limit the number of characteristics studied in the model.

**Figure 5:**
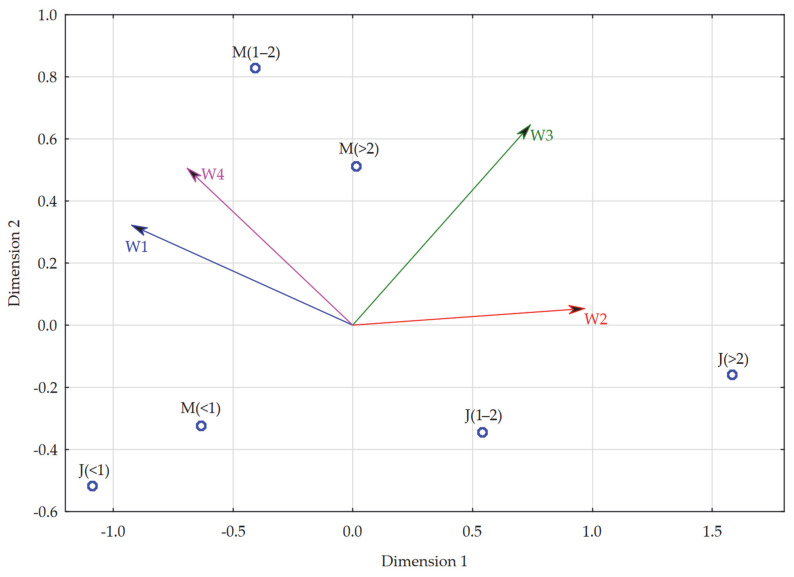
Biplot including the result of multidimensional scaling for individual objects (groups distinguished by mode of remote learning and its length) based on the various dimensions of evaluation of remote learning in higher education.

The final step in the construction of this model was the PROFIT analysis, and the result is presented in the chart below (Figure 5). This analysis showed that the dimension of negative attitudes (W4) is particularly close for those studying remotely in mixed mode for a minimum of one year (i.e., 1–2 years and more than 2 years). The socio-emotional dimension (W1) is similar for both groups, although it is less close to them (compared to W4). The socio-emotional dimension (W1), on the other hand, has the greatest importance for students studying remotely in a mixed mode for up to a year, and relatively high importance for students studying remotely in a uniform mode for the same period of time. Both of the aforementioned dimensions, which have a negative connotation in them, are definitely not significant in the evaluations of remote education for those studying remotely in a uniform mode for a minimum of one year, which focus mainly on the development dimension (W2). In addition, the time and financial dimension (W3) is relatively close for students studying remotely in a uniform mode for 1–2 years, but the most attention is paid to the above-mentioned dimension of remote learning by those studying remotely in a mixed mode for more than 2 years. Much less important is the latter dimension (W3) for those studying in a mixed mode for 1–2 years and in a uniform mode for more than 2 years. Although, taking into account the location on the multidimensional scaling map of both groups in relation to the coordinates of this dimension (feature), time and financial issues should be considered important for these students as well.

***Evaluation of Remote Learning in Higher Education in Its Various Dimensions in Relation to Gender and Length of Remote Learning***

The analysis in this section will answer the ninth research question: What dimensions are particularly important in evaluations of remote learning in higher education for men and women with different lengths of remote learning?

Another model developed concerned the evaluation of remote learning in higher education in groups distinguished by gender and length of remote learning. The methodology for building this model was analogous to the previous ones.

During the development of this model, the objects analyzed were groups of:

- Women studying remotely for up to 1 year [K(<1)],
- Women studying remotely for 1–2 years [K(1–2)],
- Women studying remotely for more than 2 years [K(>2)],
- Men studying remotely for up to 1 year [M(<1)],
- Men studying remotely for 1–2 years [M(1–2)],
- Men studying remotely for more than 2 years [M(>2)].

The variables (characteristics) were analogous to the previous model, i.e., the dimensions of evaluation of remote learning in higher education. In order to develop the model, the average scores of each dimension of evaluation of remote learning in higher education recorded in the above-mentioned groups were used. The following table presents detailed data in this regard (Table 19).

The STRESS coefficient for multidimensional scaling considering all characteristics was 0.00, indicating high reliability of the results of the multidimensional scaling procedure. The analysis of the resulting map allows us to deduce that almost every group differed from the others in evaluations of remote learning in its various dimensions. The closest to each other in terms of the above-mentioned evaluation were women studying remotely for up to a year and men, for whom this period was 1–2 years.

The regression analysis conducted for the individual dimensions of evaluation of remote learning in higher education (dependent variables) and the values of the two dimensions for each individual obtained by multidimensional scaling: Dim. 1 and Dim. 2 (explanatory variables) showed that the individual items were very well matched. The individual studied dimensions of evaluation of remote learning in higher education had a high impact on the variation of the studied units. The lowest fit was for the dimension of negative attitudes (R^2^ = 0.88), and the highest for the time and financial dimension, respectively (R^2^ = 0.99) (Table 20). Thus, again, there was no need to limit the number of characteristics studied in the model.

**Figure 6:**
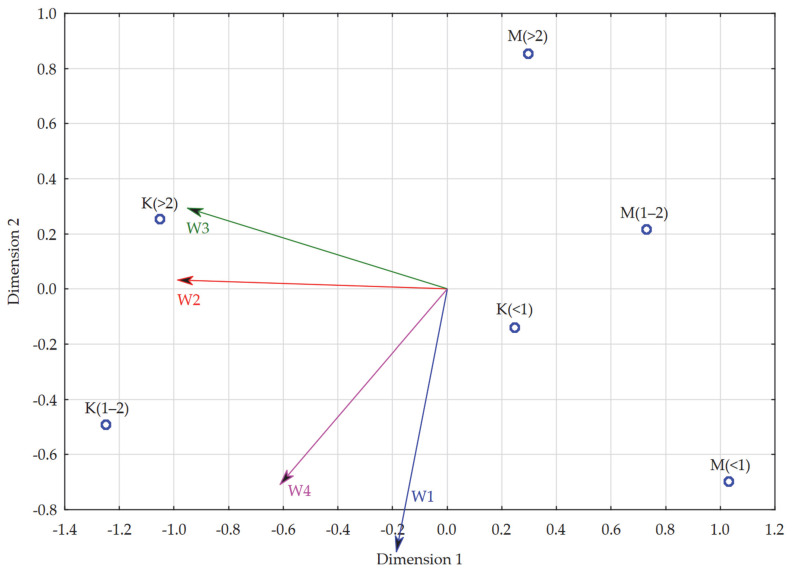
Biplot including the result of multidimensional scaling for individual objects (groups distinguished by gender and length of remote learning) based on each dimension of evaluation of remote learning in higher education.

The model developed using PROFIT analysis showed that the socio-emotional dimension (W1), as well as the negative attitude dimension (W4) were particularly important in evaluations of remote education for women studying remotely for the shortest period of time (up to a year). Women studying remotely for 1–2 years paid similar attention to the development dimension (W2) and the negative attitude dimension (W4). For women studying remotely for more than 2 years, the time and financial dimension (W3) was particularly important in their evaluation of remote education, but they also paid very high attention to the development dimension (W2). In contrast, men, regardless of the length of remote education were not particularly guided by any dimension in their evaluations. For men studying remotely for less than a year, the least attention was focused on the development (W2) and time and financial (W3) dimensions, while the greatest attention was focused on the socio-emotional (W1) dimension. For men studying remotely the longest (more than 2 years), the socio-emotional dimension (W1) and the negative attitude dimension (W4) were the least important, while the time and financial dimension (W3) was the most important. In contrast, for men studying remotely for 1–2 years, none of the dimensions was particularly important in their evaluations of remote education (Figure 6).

***Evaluation of Remote Learning in Higher Education in Its Various Dimensions in Relation to Gender and Form of Study***

The analysis in this section will answer the tenth research question: What dimensions are particularly important in evaluations of remote learning in higher education for women and men studying in different modes?

The last model developed concerned the evaluation of remote learning in higher education in groups distinguished by gender and form of study. The methodology for building this model was analogous to the previous ones.

In developing this model, the objects analyzed were groups of:

- Women studying full-time [K(S)],
- Women studying part-time [K(N)],
- Men studying full-time [M(S)],
- Men studying part-time [M(N)].

Again, the variables (characteristics) were analogous to the previous models, i.e., the dimensions of evaluation of remote learning in higher education. To develop the model, the average scores of each dimension of evaluation of remote learning in higher education recorded in the aforementioned groups were used (Table 21).

The STRESS coefficient for multidimensional scaling considering all characteristics was 0.00, which was equivalent to high reliability for the results of the multidimensional scaling procedure. From the resulting map, it can be read that almost every group differed from the others in evaluations of remote learning in its individual dimensions. The groups of women and men studying part-time were the closest to each other in terms of the aforementioned evaluation.

Regression analysis for individual dimensions of evaluation of remote learning in higher education (dependent variables) and the values of two dimensions for each unit obtained by multidimensional scaling: Dim. 1 and Dim. 2 (explanatory variables) showed a good fit between the individual units. The studied dimensions of evaluation of remote learning in higher education were characterized by a high impact on the differentiation of the studied units. The lowest fit was for the socio-emotional dimension (R^2^ = 0.70), while the highest-for the development dimension respectively (R^2^ = 0.99) (Table 22). This again meant there was no need to limit the number of characteristics studied in the model.

**Figure 7:**
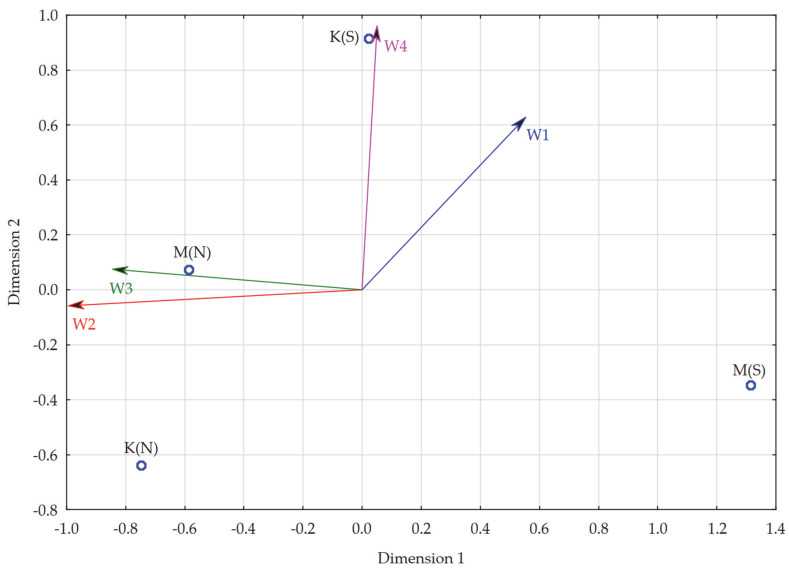
Biplot including the result of multidimensional scaling for individual objects (groups distinguished by gender and form of study) based on each dimension of evaluation of remote learning in higher education.

The model developed using PROFIT analysis showed that men studying part-time focused particularly on the time and financial (W3) and development (W2) dimensions in their evaluations of remote learning. Both of the aforementioned dimensions were of relatively high (though lesser) importance for women studying part-time. At the same time, it is worth noting that for the latter group, its negative dimensions were definitely not important in evaluations of remote learning. In contrast, full-time students paid more attention to the negative sides of remote learning, with women particularly close to the dimension of negative attitudes (W4), i.e., potential threats. Male full-time students were closest in their evaluations of remote learning to the socio-emotional dimension (W1), which was nevertheless even closer to female full-time students. In contrast, the development (W2) and time and financial (W3) dimensions were decidedly unimportant for men studying full-time; for women studying in this way, the closest of the two aforementioned dimensions was time and financial (W3), although it was much less important compared to dimensions with negative connotations (Figure 7).

## 5. Conclusions

Students express a positive view of remote learning and consider it to be as effective as on-site education. Among the advantages of remote learning for students, the most important are saving time, the ability to study at a university far from home (another city, another country), the ability to combine work and study, and the reduction in commuting costs.

The disadvantages of remote learning of greatest importance to students include the loss of social ties due to lack of contact with peers, feelings of fatigue resulting from excessive use of information and communication tools, and greater susceptibility to various forms of distraction.

Remote education in higher education is evaluated by students in socio-emotional, development, time and financial, and negative attitude dimensions. The shape of students’ education is relevant to each dimension of their evaluation of remote learning in higher education. The socio-emotional dimension of remote learning is of greater importance to students who study remotely in a mixed mode (compared to uniform). The development dimension is particularly important for students who participate in remote learning activities longer in the day and for a longer period of time. Moreover, a longer period of remote learning promotes a greater importance of the time and financial dimension in the evaluation of remote learning. In addition, full-time students are more likely to focus their attention on the negative sides of remote learning (the socio-emotional dimension and the negative attitude dimension), while part-time students are more likely to focus on the positive sides (the development dimension and the time and financial dimension).

The importance of the development and time and financial dimensions in evaluations of remote learning differs between female and male students. Women are more likely to focus attention on the above-mentioned positive dimensions of remote learning.

Dimensions with negative connotations for evaluation of remote learning are more significant for full-time students and those studying remotely for a minimum of more than a year and part-time students and those studying remotely for less than a year. Positive dimensions of remote learning are more often perceived by part-time students and those studying remotely for a period of 1–2 years.

Negative dimensions of evaluations of remote learning in higher education are perceived mainly by those studying remotely in a mixed mode for up to 2 years and in a uniform mode for up to a year. The positive dimensions of the above-mentioned evaluations are mainly relevant to those studying remotely in a uniform mode for a minimum of one year. In contrast, students studying remotely in a mixed mode for more than 2 years pay similarly high attention to both positive issues (especially the time and financial dimension) and negative issues (especially negative attitudes).

The development dimension and the negative attitude dimension are of high importance for women studying remotely for 1–2 years. Also, women studying remotely for more than 2 years pay special attention to the development dimension, but also to the time and financial dimension. Negative dimensions of evaluation of remote learning in higher education (socio-emotional and negative attitudes) are important for women studying remotely for the shortest period of time. In contrast, men, regardless of the duration of remote learning, do not pay particular attention to any of the dimensions in their evaluations of remote learning.

Dimensions with negative connotations (socio-emotional and negative-attitude) are particularly important for women and men studying full-time, while dimensions with positive connotations (development and time-financial) are especially important for part-time students, respectively.

As mentioned earlier, the survey results show that remote learning positively appeals to Polish university students. Due to the mentioned significant benefits of this form of education, a hybrid mode could be introduced at Polish universities, allowing for equally effective acquisition of knowledge as in the case of stationary learning. It would also incentivize potential candidates to study in selected fields. It is worth noting that awareness of the main barriers (such as the feeling of fatigue resulting from excessive use of information and communication tools) will allow rational planning of remote learning.

The manuscript has some limitations. Firstly, the research was conducted only in higher education. Secondly was conducted only in Poland. There may be differences in perceptions of remote learning depending on the levels of education (elementary school, high school, university), which creates implications for future comparative research. The authors’ intention at this stage of the research was to diagnose potential differences and correlations in the perception of advantages and disadvantages of remote education with other factors related to gender and the form, mode, and length of education. The results obtained encourage further exploration of this topic, with the possibility of separating out, for example, different fields of study and/or making comparisons across countries. 

## Figures and Tables

**Table 1:** Age of respondents in total and by gender.

		Total (n = 999)		Gender				Mann-Whitney U Test	r_g_
				Women (n = 518)		Men (n = 481)			
		n	%	n	%	n	%		
Age (in years)	19–25	829	82.98%	430	83.01%	399	82.95%	Z = −0.14; *p* = 0.887	−0.01
	26–32	70	7.01%	43	8.30%	27	5.61%		
	33 and more	100	10.01%	45	8.69%	55	11.43%		

**Table 2:** Education of respondents in total and by gender.

		Total (n = 999)		Gender				Mann-Whitney U Test/χ^2^	r_g/_φ
				Women (n = 518)		Men (n = 481)			
		n	%	n	%	n	%		
Length of remote learning	≤1 year	232	23.22%	110	21.24%	122	25.36%	Z = 2.6; *p* < 0.01	0.10
	1–2 years	533	53.35%	266	51.35%	267	55.51%		
	>2 years	234	23.42%	142	27.41%	92	19.13%		
Form of studies	Full-time	766	76.68%	387	74.71%	379	78.79%	χ^2^(1) = 2.33; *p* = 0.127	−0.05
	Part-time	233	23.32%	131	25.29%	102	21.21%		
Level of studies	1st degree	787	78.78%	413	79.73%	374	77.75%	χ^2^(1) = 0.58; *p* = 0.446	0.02
	2nd degree	212	21.22%	105	20.27%	107	22.25%		
University	State	840	84.08%	419	80.89%	421	87.53%	χ^2^(1) = 8.21; *p* < 0.01	−0.09
	Private	159	15.92%	99	19.11%	60	12.47%		

**Table 3:** Degree of significance to respondents of the various advantages of remote learning.

Advantages of Remote Learning	Degree of Significance									
	They Are Not an Advantage		They Are a Minimal Advantage		They Are a Moderate Advantage		They Are a Significant Advantage		They Are a Maximum Advantage	
	n	%	n	%	n	%	n	%	n	%
Time saving	28	2.80%	26	2.60%	108	10.81%	197	19.72%	640	64.06%
Flexible study time	45	4.50%	53	5.31%	175	17.52%	281	28.13%	445	44.54%
Ability to adjust the pace of learning to one’ s needs	87	8.71%	104	10.41%	215	21.52%	242	24.22%	351	35.14%
Ability to study in a university far from the place of residence (another city, another country)	22	2.20%	24	2.40%	80	8.01%	121	12.11%	752	75.28%
Quick acquisition of knowledge	113	11.31%	161	16.12%	305	30.53%	223	22.32%	197	19.72%
Opportunity to learn about information and communication tools	84	8.41%	125	12.51%	264	26.43%	253	25.33%	273	27.33%
Opportunity to combine work and study	50	5.01%	43	4.30%	131	13.11%	207	20.72%	568	56.86%
Reduction in commuting costs	13	1.30%	17	1.70%	52	5.21%	102	10.21%	815	81.58%
Development of digital competencies	67	6.71%	98	9.81%	256	25.63%	224	22.42%	354	35.44%

**Table 4:** Evaluation of the importance for the respondents of the individual advantages of remote learning (on a point scale of 1–5).

	Descriptive Statistics					
	Mean ± Stand. Dev.	Median (Q25–Q75)	Min.–Max.	Confidence Interval		Stand. Error
				−95.00%	+95.00%	
Time saving	4.4 ± 0.97	5 (4–5)	1–5	4.34	4.46	0.03
Flexible study time	4.03 ± 1.11	4 (3–5)	1–5	3.96	4.10	0.04
Ability to adjust the pace of learning to one’ s needs	3.67 ± 1.29	4 (3–5)	1–5	3.59	3.75	0.04
Ability to study in a university far from the place of residence (another city, another country)	4.56 ± 0.9	5 (5–5)	1–5	4.50	4.61	0.03
Quick acquisition of knowledge	3.23 ± 1.25	3 (2–4)	1–5	3.15	3.31	0.04
Opportunity to learn about information and communication tools	3.51 ± 1.25	4 (3–5)	1–5	3.43	3.58	0.04
Opportunity to combine work and study	4.2 ± 1.13	5 (4–5)	1–5	4.13	4.27	0.04
Reduction in commuting costs	4.69 ± 0.76	5 (5–5)	1–5	4.64	4.74	0.02
Development of digital competencies	3.7 ± 1.23	4 (3–5)	1–5	3.62	3.78	0.04

**Table 5:** Cluster elements for the analyzed advantages of remote learning (results of cluster analysis using k-means clustering).

Elements of Individual Clusters		Distance	Descriptive Statistics of the Factors Included in Each Cluster					
			Mean ± Stand. Dev.	Median (Q25–Q75)	Min.–Max.	Confidence Interval		Stand. Error
						−95.00%	+95.00%	
Cluster no. 1	Flexible study time	0.5535	3.85 ± 1.22	4 (3–5)	1–5	3.79	3.90	0.03
	Ability to adapt the pace of learning to one’s needs	0.5535						
Cluster no. 2	Quick acquisition of knowledge	0.7875	3.48 ± 1.26	4 (3–5)	1–5	3.43	3.52	0.02
	Opportunity to learn about information and communication tools	0.5836						
	Development of digital competencies	0.6768						
Cluster no. 3	Time saving	0.6837	4.46 ± 0.97	5 (4–5)	1–5	4.43	4.49	0.02
	Opportunity to study in a university far from the place of residence (other city, other country)	0.5969						
	Opportunity to combine work and study	0.7457						
	Reduction in commuting costs	0.5434						

**Table 6:** The degree of significance for the respondents of the individual disadvantages of remote learning.

Disadvantages of Remote Learning	Degree of Significance									
	They Are Not a Disadvantage		They Are a Minimal Disadvantage		They Are a Moderate Disadvantage		They Are a Significant Disadvantage		They Are a Maximum Disadvantage	
	n	%	n	%	n	%	n	%	n	%
Technical problems during remote classes	104	10.41%	252	25.23%	324	32.43%	208	20.82%	111	11.11%
Feeling of isolation/alienation	291	29.13%	191	19.12%	191	19.12%	163	16.32%	163	16.32%
Loss of social ties due to lack of contact with peers	186	18.62%	161	16.12%	195	19.52%	224	22.42%	233	23.32%
Lack of sufficient lecturer-student interaction	184	18.42%	229	22.92%	254	25.43%	214	21.42%	118	11.81%
Feeling of fatigue due to excessive use of information and communication tools	212	21.22%	177	17.72%	196	19.62%	190	19.02%	224	22.42%
Insufficient preparation of lecturers to teach remotely	260	26.03%	226	22.62%	262	26.23%	156	15.62%	95	9.51%
Fear of change	508	50.85%	200	20.02%	171	17.12%	69	6.91%	51	5.11%
Possibility of Internet addiction	516	51.65%	197	19.72%	147	14.71%	78	7.81%	61	6.11%
Greater susceptibility to various forms of distraction	199	19.92%	144	14.41%	228	22.82%	218	21.82%	210	21.02%

**Table 7:** Rating of the significance to respondents of the individual disadvantages of remote education (on a point scale of 1–5).

	Descriptive Statistics					
	Mean ± Stand. Dev.	Median (Q25–Q75)	Min.–Max.	Confidence Interval		Stand. Error
				−95.00%	+95.00%	
Technical problems during remote classes	2.97 ± 1.15	3 (2–4)	1–5	2.90	3.04	0.04
Feeling of isolation/alienation	2.72 ± 1.45	3 (1–4)	1–5	2.63	2.81	0.05
Loss of social ties due to lack of contact with peers	3.16 ± 1.43	3 (2–4)	1–5	3.07	3.25	0.05
Lack of sufficient lecturer-student interaction	2.85 ± 1.28	3 (2–4)	1–5	2.77	2.93	0.04
Feeling of fatigue due to excessive use of information and communication tools	3.04 ± 1.45	3 (2–4)	1–5	2.95	3.13	0.05
Insufficient preparation of lecturers to teach remotely	2.6 ± 1.28	3 (1–4)	1–5	2.52	2.68	0.04
Fear of change	1.95 ± 1.19	1 (1–3)	1–5	1.88	2.03	0.04
Possibility of Internet addiction	1.97 ± 1.24	1 (1–3)	1–5	1.89	2.05	0.04
Greater susceptibility to various forms of distraction	3.1 ± 1.41	3 (2–4)	1–5	3.01	3.18	0.04

**Table 8:** Cluster elements for the analyzed disadvantages of remote learning (results of cluster analysis using k-means clustering).

Elements of Individual Clusters		Distance	Descriptive Statistics of the Factors Included in Each Cluster					
			Mean ± Stand. Dev.	Median (Q25–Q75)	Min.–Max.	Confidence Interval		Stand. Error
						−95.00%	+95.00%	
Cluster no. 1	Feeling of isolation/alienation	0.7913	2.97 ± 1.41	3 (2–4)	1–5	2.93	3.01	0.02
	Loss of social ties due to lack of contact with peers	0.7802						
	Lack of sufficient lecturer-student interaction	0.7909						
	Feeling of fatigue due to excessive use of information and communication tools	0.8626						
	Greater susceptibility to various forms of distraction	0.9169						
Cluster no. 2	Technical problems during remote classes	0.6848	2.78 ± 1.23	3 (2–4)	1–5	2.73	2.84	0.03
	Insufficient preparation of lecturers to teach remotely	0.6848						
Cluster no. 3	Fear of change	0.5795	1.96 ± 1.21	1 (1–3)	1–5	1.91	2.02	0.03
	Possibility of Internet addiction	0.5795						

**Table 9:** Correlations between items determining evaluation of remote learning in higher education-part 1.

n = 999	Pearson’s Linear Correlation								
	Item 1	Item 2	Item 3	Item 4	Item 5	Item 6	Item 7	Item 8	Item 9
Item 1	–	r = 0.5; *p* < 0.001	r = 0.4; *p* < 0.001	r = 0.35; *p* < 0.001	r = 0.36; *p* < 0.001	r = 0.27; *p* < 0.001	r = 0.4; *p* < 0.001	r = 0.41; *p* < 0.001	r = 0.32; *p* < 0.001
Item 2	r = 0.5; *p* < 0.001	–	r = 0.63; *p* < 0.001	r = 0.31; *p* < 0.001	r = 0.46; *p* < 0.001	r = 0.32; *p* < 0.001	r = 0.42; *p* < 0.001	r = 0.32; *p* < 0.001	r = 0.36; *p* < 0.001
Item 3	r = 0.4; *p* < 0.001	r = 0.63; *p* < 0.001	–	r = 0.32; *p* < 0.001	r = 0.55; *p* < 0.001	r = 0.4; *p* < 0.001	r = 0.42; *p* < 0.001	r = 0.28; *p* < 0.001	r = 0.44; *p* < 0.001
Item 4	r = 0.35; *p* < 0.001	r = 0.31; *p* < 0.001	r = 0.32; *p* < 0.001	–	r = 0.31; *p* < 0.001	r = 0.38; *p* < 0.001	r = 0.45; *p* < 0.001	r = 0.56; *p* < 0.001	r = 0.37; *p* < 0.001
Item 5	r = 0.36; *p* < 0.001	r = 0.46; *p* < 0.001	r = 0.55; *p* < 0.001	r = 0.31; *p* < 0.001	–	r = 0.56; *p* < 0.001	r = 0.46; *p* < 0.001	r = 0.24; *p* < 0.001	r = 0.48; *p* < 0.001
Item 6	r = 0.27; *p* < 0.001	r = 0.32; *p* < 0.001	r = 0.4; *p* < 0.001	r = 0.38; *p* < 0.001	r = 0.56; *p* < 0.001	–	r = 0.41; *p* < 0.001	r = 0.32; *p* < 0.001	r = 0.69; *p* < 0.001
Item 7	r = 0.4; *p* < 0.001	r = 0.42; *p* < 0.001	r = 0.42; *p* < 0.001	r = 0.45; *p* < 0.001	r = 0.46; *p* < 0.001	r = 0.41; *p* < 0.001	–	r = 0.51; *p* < 0.001	r = 0.47; *p* < 0.001
Item 8	r = 0.41; *p* < 0.001	r = 0.32; *p* < 0.001	r = 0.28; *p* < 0.001	r = 0.56; *p* < 0.001	r = 0.24; *p* < 0.001	r = 0.32; *p* < 0.001	r = 0.51; *p* < 0.001	–	r = 0.37; *p* < 0.001
Item 9	r = 0.32; *p* < 0.001	r = 0.36; *p* < 0.001	r = 0.44; *p* < 0.001	r = 0.37; *p* < 0.001	r = 0.48; *p* < 0.001	r = 0.69; *p* < 0.001	r = 0.47; *p* < 0.001	r = 0.37; *p* < 0.001	–
Item 10	r = −0.12; *p* < 0.001	r = −0.19; *p* < 0.001	r = −0.25; *p* < 0.001	r = −0.09; *p* < 0.01	r = −0.33; *p* < 0.001	r = −0.25; *p* < 0.001	r = −0.16; *p* < 0.001	r = −0.07; *p* < 0.05	r = −0.23; *p* < 0.001
Item 11	r = −0.24; *p* < 0.001	r = −0.31; *p* < 0.001	r = −0.36; *p* < 0.001	r = −0.13; *p* < 0.001	r = −0.41; *p* < 0.001	r = −0.21; *p* < 0.001	r = −0.26; *p* < 0.001	r = −0.15; *p* < 0.001	r = −0.21; *p* < 0.001
Item 12	r = −0.23; *p* < 0.001	r = −0.3; *p* < 0.001	r = −0.34; *p* < 0.001	r = −0.12; *p* < 0.001	r = −0.4; *p* < 0.001	r = −0.25; *p* < 0.001	r = −0.22; *p* < 0.001	r = −0.14; *p* < 0.001	r = −0.23; *p* < 0.001
Item 13	r = −0.25; *p* < 0.001	r = −0.3; *p* < 0.001	r = −0.4; *p* < 0.001	r = −0.16; *p* < 0.001	r = −0.45; *p* < 0.001	r = −0.29; *p* < 0.001	r = −0.29; *p* < 0.001	r = −0.18; *p* < 0.001	r = −0.28; *p* < 0.001
Item 14	r = −0.23; *p* < 0.001	r = −0.3; *p* < 0.001	r = −0.33; *p* < 0.001	r = −0.12; *p* < 0.001	r = −0.4; *p* < 0.001	r = −0.24; *p* < 0.001	r = −0.28; *p* < 0.001	r = −0.12; *p* < 0.001	r = −0.24; *p* < 0.001
Item 15	r = −0.15; *p* < 0.001	r = −0.12; *p* < 0.001	r = −0.18; *p* < 0.001	r = −0.13; *p* < 0.001	r = −0.31; *p* < 0.001	r = −0.25; *p* < 0.001	r = −0.17; *p* < 0.001	r = −0.12; *p* < 0.001	r = −0.19; *p* < 0.001
Item 16	r = −0.19; *p* < 0.001	r = −0.19; *p* < 0.001	r = −0.18; *p* < 0.001	r = −0.15; *p* < 0.001	r = −0.19; *p* < 0.001	r = −0.09; *p* < 0.01	r = −0.16; *p* < 0.001	r = −0.17; *p* < 0.001	r = −0.06; *p* < 0.05
Item 17	r = −0.2; *p* < 0.001	r = −0.21; *p* < 0.001	r = −0.21; *p* < 0.001	r = −0.07; *p* < 0.05	r = −0.27; *p* < 0.001	r = −0.11; *p* < 0.01	r = −0.18; *p* < 0.001	r = −0.16; *p* < 0.001	r = −0.12; *p* < 0.001
Item 18	r = −0.19; *p* < 0.001	r = −0.27; *p* < 0.001	r = −0.33; *p* < 0.001	r = −0.13; *p* < 0.001	r = −0.43; *p* < 0.001	r = −0.28; *p* < 0.001	r = −0.25; *p* < 0.001	r = −0.13; *p* < 0.001	r = −0.3; *p* < 0.001

**Table 10:** Correlations between items determining evaluation of remote learning in higher education-part 2.

n = 999	Pearson’s Linear Correlation								
	Item 10	Item 11	Item 12	Item 13	Item 14	Item 15	Item 16	Item 17	Item 18
Item 1	r = −0.12; *p* < 0.001	r = −0.24; *p* < 0.001	r = −0.23; *p* < 0.001	r = −0.25; *p* < 0.001	r = −0.23; *p* < 0.001	r = −0.15; *p* < 0.001	r = −0.19; *p* < 0.001	r = −0.2; *p* < 0.001	r = −0.19; *p* < 0.001
Item 2	r = −0.19; *p* < 0.001	r = −0.31; *p* < 0.001	r = −0.3; *p* < 0.001	r = −0.3; *p* < 0.001	r = −0.3; *p* < 0.001	r = −0.12; *p* < 0.001	r = −0.19; *p* < 0.001	r = −0.21; *p* < 0.001	r = −0.27; *p* < 0.001
Item 3	r = −0.25; *p* < 0.001	r = −0.36; *p* < 0.001	r = −0.34; *p* < 0.001	r = −0.4; *p* < 0.001	r = −0.33; *p* < 0.001	r = −0.18; *p* < 0.001	r = −0.18; *p* < 0.001	r = −0.21; *p* < 0.001	r = −0.33; *p* < 0.001
Item 4	r = −0.09; *p* < 0.01	r = −0.13; *p* < 0.001	r = −0.12; *p* < 0.001	r = −0.16; *p* < 0.001	r = −0.12; *p* < 0.001	r = −0.13; *p* < 0.001	r = −0.15; *p* < 0.001	r = −0.07; *p* < 0.05	r = −0.13; *p* < 0.001
Item 5	r = −0.33; *p* < 0.001	r = −0.41; *p* < 0.001	r = −0.4; *p* < 0.001	r = −0.45; *p* < 0.001	r = −0.4; *p* < 0.001	r = −0.31; *p* < 0.001	r = −0.19; *p* < 0.001	r = −0.27; *p* < 0.001	r = −0.43; *p* < 0.001
Item 6	r = −0.25; *p* < 0.001	r = −0.21; *p* < 0.001	r = −0.25; *p* < 0.001	r = −0.29; *p* < 0.001	r = −0.24; *p* < 0.001	r = −0.25; *p* < 0.001	r = −0.09; *p* < 0.01	r = −0.11; *p* < 0.01	r = −0.28; *p* < 0.001
Item 7	r = −0.16; *p* < 0.001	r = −0.26; *p* < 0.001	r = −0.22; *p* < 0.001	r = −0.29; *p* < 0.001	r = −0.28; *p* < 0.001	r = −0.17; *p* < 0.001	r = −0.16; *p* < 0.001	r = −0.18; *p* < 0.001	r = −0.25; *p* < 0.001
Item 8	r = −0.07; *p* < 0.05	r = −0.15; *p* < 0.001	r = −0.14; *p* < 0.001	r = −0.18; *p* < 0.001	r = −0.12; *p* < 0.001	r = −0.12; *p* < 0.001	r = −0.17; *p* < 0.001	r = −0.16; *p* < 0.001	r = −0.13; *p* < 0.001
Item 9	r = −0.23; *p* < 0.001	r = −0.21; *p* < 0.001	r = −0.23; *p* < 0.001	r = −0.28; *p* < 0.001	r = −0.24; *p* < 0.001	r = −0.19; *p* < 0.001	r = −0.06; *p* < 0.05	r = −0.12; *p* < 0.001	r = −0.3; *p* < 0.001
Item 10	–	r = 0.42; *p* < 0.001	r = 0.4; *p* < 0.001	r = 0.42; *p* < 0.001	r = 0.46; *p* < 0.001	r = 0.42; *p* < 0.001	r = 0.3; *p* < 0.001	r = 0.32; *p* < 0.001	r = 0.4; *p* < 0.001
Item 11	r = 0.42; *p* < 0.001	–	r = 0.77; *p* < 0.001	r = 0.58; *p* < 0.001	r = 0.6; *p* < 0.001	r = 0.32; *p* < 0.001	r = 0.39; *p* < 0.001	r = 0.43; *p* < 0.001	r = 0.54; *p* < 0.001
Item 12	r = 0.4; *p* < 0.001	r = 0.77; *p* < 0.001	–	r = 0.6; *p* < 0.001	r = 0.55; *p* < 0.001	r = 0.3; *p* < 0.001	r = 0.33; *p* < 0.001	r = 0.37; *p* < 0.001	r = 0.54; *p* < 0.001
Item 13	r = 0.42; *p* < 0.001	r = 0.58; *p* < 0.001	r = 0.6; *p* < 0.001	–	r = 0.6; *p* < 0.001	r = 0.42; *p* < 0.001	r = 0.35; *p* < 0.001	r = 0.38; *p* < 0.001	r = 0.51; *p* < 0.001
Item 14	r = 0.46; *p* < 0.001	r = 0.6; *p* < 0.001	r = 0.55; *p* < 0.001	r = 0.6; *p* < 0.001	–	r = 0.37; *p* < 0.001	r = 0.36; *p* < 0.001	r = 0.44; *p* < 0.001	r = 0.54; *p* < 0.001
Item 15	r = 0.42; *p* < 0.001	r = 0.32; *p* < 0.001	r = 0.3; *p* < 0.001	r = 0.42; *p* < 0.001	r = 0.37; *p* < 0.001	–	r = 0.38; *p* < 0.001	r = 0.34; *p* < 0.001	r = 0.39; *p* < 0.001
Item 16	r = 0.3; *p* < 0.001	r = 0.39; *p* < 0.001	r = 0.33; *p* < 0.001	r = 0.35; *p* < 0.001	r = 0.36; *p* < 0.001	r = 0.38; *p* < 0.001	–	r = 0.54; *p* < 0.001	r = 0.37; *p* < 0.001
Item 17	r = 0.32; *p* < 0.001	r = 0.43; *p* < 0.001	r = 0.37; *p* < 0.001	r = 0.38; *p* < 0.001	r = 0.44; *p* < 0.001	r = 0.34; *p* < 0.001	r = 0.54; *p* < 0.001	–	r = 0.47; *p* < 0.001
Item 18	r = 0.4; *p* < 0.001	r = 0.54; *p* < 0.001	r = 0.54; *p* < 0.001	r = 0.51; *p* < 0.001	r = 0.54; *p* < 0.001	r = 0.39; *p* < 0.001	r = 0.37; *p* < 0.001	r = 0.47; *p* < 0.001	–

**Table 11:** Eigenvalue results (Kaiser-Gutman criterion).

Factor	Eigenvalue	% of Total Variance	Cumulative Eigenvalue	Cumulative % of Total Variance
1	6.5180	36.21	6.5180	36.21
2	2.5095	13.94	9.0276	50.15
3	1.2246	6.80	10.2522	56.96
4	1.0865	6.04	11.3387	62.99
5	0.8370	4.65	12.1757	67.64
6	0.7007	3.89	12.8764	71.54
7	0.5913	3.29	13.4677	74.82
8	0.5753	3.20	14.0430	78.02
9	0.5611	3.12	14.6041	81.13
10	0.4881	2.71	15.0922	83.85
11	0.4656	2.59	15.5579	86.43
12	0.4554	2.53	16.0132	88.96
13	0.4326	2.40	16.4458	91.37
14	0.3937	2.19	16.8395	93.55
15	0.3525	1.96	17.1920	95.51
16	0.3219	1.79	17.5139	97.30
17	0.2681	1.49	17.7819	98.79
18	0.2181	1.21	18.0000	100.00

**Table 12:** Results of exploratory factor analysis (principal component method) for the evaluation of remote learning in higher education.

	Factor			
	1	2	3	4
Item 1	−0.25	0.08	**0.70**	0.02
Item 2	−0.45	0.17	**0.62**	0.26
Item 3	−0.50	0.34	0.47	0.24
Item 4	0.10	0.36	**0.65**	−0.23
Item 5	−0.48	**0.58**	0.27	0.04
Item 6	−0.13	**0.84**	0.20	−0.04
Item 7	−0.15	0.41	**0.61**	−0.09
Item 8	0.09	0.24	**0.74**	−0.28
Item 9	−0.13	**0.79**	0.29	0.00
Item 10	0.50	−0.33	0.13	0.34
Item 11	**0.81**	−0.06	−0.12	0.15
Item 12	**0.80**	−0.11	−0.08	0.10
Item 13	**0.71**	−0.23	−0.09	0.21
Item 14	**0.72**	−0.17	−0.06	0.24
Item 15	0.31	−0.33	0.07	**0.61**
Item 16	0.38	0.14	−0.24	**0.66**
Item 17	0.49	0.10	−0.19	**0.55**
Item 18	**0.65**	−0.24	−0.03	0.30
% of variance explained	23.83%	14.28%	15.37%	9.52%
Cronbach’s α	α = 0.87	α = 0.80	α = 0.78	α = 0.68

The range of explained variance is 62.99%. Factor loadings meeting the inclusion criterion for a factor are marked in bold.

**Table 13:** Dimensions of remote learning evaluation in higher education (descriptive statistics).

	Descriptive Statistics					
	Mean ± Stand. Dev.	Median (Q25–Q75)	Min.–Max.	Confidence Interval		Stand. Error
				−95.00%	+95.00%	
Socio-emotional dimension	2.97 ± 1.15	3 (2–4)	1–5	2.90	3.04	0.04
Development dimension	3.48 ± 1.05	3.67 (2.67–4.33)	1–5	3.41	3.54	0.03
Time and financial dimension	4.38 ± 0.72	4.6 (4–5)	1–5	4.33	4.42	0.02
Dimension of negative attitude	2.17 ± 0.97	2 (1.33–2.67)	1–5	2.11	2.23	0.03

**Table 14:** Relationship between respondents’ gender and their evaluation of remote learning in higher education across dimensions.

	Gender	Descriptive Statistics						Mann-Whitney U test	r_g_
		Mean ± Stand. Dev.	Median (Q25–Q75)	Min.–Max.	Confidence Interval		Stand. Error		
					−95.00%	+95.00%			
Socio-emotional dimension	Women (n = 518)	3.03 ± 1.18	3 (2–4)	1–5	2.93	3.13	0.05	Z = 1.6; *p* = 0.11	0.06
	Men (n = 481)	2.91 ± 1.11	2.8 (2–3.8)	1–5	2.81	3.01	0.05		
Development dimension	Women (n = 518)	3.65 ± 1.02	3.67 (3–4.67)	1–5	3.56	3.73	0.04	Z = 5.11; *p* < 0.001	0.19
	Men (n = 481)	3.3 ± 1.07	3.33 (2.67–4)	1–5	3.20	3.40	0.05		
Time and financial dimension	Women (n = 518)	4.49 ± 0.65	4.6 (4.2–5)	1–5	4.43	4.54	0.03	Z = 5.27; *p* < 0.001	0.19
	Men (n = 481)	4.26 ± 0.77	4.4 (4–4.8)	1–5	4.19	4.32	0.03		
Negative attitude dimension	Women (n = 518)	2.25 ± 1.03	2 (1.33–3)	1–5	2.16	2.34	0.05	Z = 1.69; *p* < 0.09	0.06
	Men (n = 481)	2.1 ± 0.89	2 (1.33–2.67)	1–5	2.02	2.18	0.04		

**Table 15:** Average evaluation of remote learning in higher education in each of its dimensions in groups distinguished by form of study and length of remote learning.

	Socio-Emotional Dimension [W1]	Development Dimension [W2]	Time and Financial Dimension [W3]	Negative Attitude Dimension [W4]
Full-time/≤ 1 year [S(<1)]	3.07	3.25	4.19	2.18
Full-time/1–2 years [S(1–2)]	3.04	3.42	4.35	2.22
Full-time/> 2 years [S(>2)]	2.97	3.44	4.51	2.19
Part-time/≤ 1 year [N(<1)]	2.89	3.52	4.36	2.15
Part-time/1–2 years [N(1–2)]	2.84	3.86	4.54	2.14
Part-time/> 2 years [N(>2)]	2.56	3.88	4.43	1.95

**Table 16:** The results of the regression analysis between the individual dimensions of evaluation of remote learning in higher education and the obtained from the regression analysis dimensions of the studied units (for groups distinguished by the form of study and length of remote learning).

	Absolute Term		Dim. 1		Dim. 2		R^2^
	b0	*p*	b	*p*	b	*p*	
Socio-emotional dimension (W1)	2.896	*p* < 0.001	−0.169	*p* < 0.05	0.084	*p* = 0.394	0.86
Development dimension (W2)	3.559	*p* < 0.001	0.252	*p* < 0.001	0.074	*p* < 0.098	0.99
Time and financial dimension (W3)	4.396	*p* < 0.001	0.082	*p* < 0.05	0.191	*p* < 0.05	0.88
Negative attitude dimension (W4)	2.137	*p* < 0.001	−0.078	*p* < 0.05	0.103	*p* < 0.071	0.90

**Table 17:** Average evaluation of remote learning in higher education in its various dimensions in groups distinguished by the mode of remote learning and its length.

	Socio-Emotional Dimension [W1]	Development Dimension [W2]	Time and Financial Dimension [W3]	Negative Attitude Dimension [W4]
Uniform/≤1 year [J(<1)]	3.08	3.24	4.21	2.24
Uniform/1–2 years [J(1–2)]	2.86	3.58	4.36	2.11
Uniform/>2 years [J(>2)]	2.73	3.78	4.50	2.06
Mixed/≤1 year [M(<1)]	2.99	3.37	4.23	2.10
Mixed/1–2 years [M(1–2)]	3.12	3.46	4.43	2.28
Mixed/>2 years [M(>2)]	2.95	3.40	4.47	2.17

**Table 18:** The results of the regression analysis between the individual dimensions of the evaluation of remote learning in higher education and the resulting dimensions of the studied units (for groups distinguished by the mode of remote learning and its length).

	Absolute Term		Dim. 1		Dim. 2		R^2^
	b0	*p*	b	*p*	b	*p*	
Socio-emotional dimension (W1)	2.956	*p* < 0.001	−0.140	*p* < 0.01	0.086	*p* < 0.083	0.95
Development dimension (W2)	3.470	*p* < 0.001	0.190	*p* < 0.01	0.018	*p* = 0.739	0.94
Time and financial dimension (W3)	4.366	*p* < 0.001	0.096	*p* < 0.01	0.147	*p* < 0.01	0.96
Negative attitude dimension (W4)	2.160	*p* < 0.001	−0.061	*p* = 0.104	0.079	*p* = 0.19	0.73

**Table 19:** Average evaluation of remote learning in higher education in each of its dimensions in groups distinguished by gender and length of remote learning.

	Socio-Emotional Dimension [W1]	Development Dimension [W2]	Time and Financial Dimension [W3]	Negative Attitude Dimension [W4]
Women/≤1 year [K(<1)]	3.03	3.43	4.30	2.16
Women/1–2 years [K(1–2)]	3.09	3.71	4.54	2.33
Women/>2 years [K(>2)]	2.91	3.69	4.53	2.15
Men/≤1 year [M(<1)]	3.04	3.18	4.15	2.18
Men/1–2 years [M(1–2)]	2.90	3.33	4.25	2.07
Men/>2 years [M(>2)]	2.76	3.36	4.41	2.08

**Table 20:** The results of the regression analysis between the individual dimensions of the evaluation of remote learning in higher education and the obtained from the regression analysis dimensions of the studied units (for groups distinguished by gender and length of remote learning).

	Absolute Term		Dim. 1		Dim. 2		R^2^
	b0	*p*	b	*p*	b	*p*	
Socio-emotional dimension (W1)	2.957	*p* < 0.001	−0.024	*p* = 0.283	−0.204	*p* < 0.01	0.94
Development dimension (W2)	3.451	*p* < 0.001	−0.220	*p* < 0.01	0.012	*p* = 0.757	0.97
Time and financial dimension (W3)	4.363	*p* < 0.001	−0.159	*p* < 0.001	0.082	*p* < 0.05	0.99
Negative attitude dimension (W4)	2.161	*p* < 0.001	−0.062	*p* < 0.057	−0.120	*p* < 0.05	0.88

**Table 21:** Average evaluation of remote learning in higher education in each of its dimensions in groups distinguished by gender and form of study.

	Socio-Emotional Dimension [W1]	Development Dimension [W2]	Time and Financial Dimension [W3]	Negative Attitude Dimension [W4]
Women/Full-time [K(S)]	3.10	3.57	4.46	2.31
Women/Part-time [K(N)]	2.80	3.85	4.55	2.07
Men/Full-time [M(S)]	2.96	3.19	4.22	2.09
Men/Part-time [M(N)]	2.73	3.72	4.38	2.11

**Table 22:** The results of the regression analysis between the individual dimensions of evaluation of remote learning in higher education and the dimensions of the surveyed units (for groups distinguished by gender and form of study) obtained from the regression analysis.

	Absolute Term		Dim. 1		Dim. 2		R^2^
	b0	*p*	b	*p*	b	*p*	
Socio-emotional dimension (W1)	2.898	*p* < 0.05	0.100	*p* = 0.497	0.157	*p* = 0.456	0.70
Development dimension (W2)	3.585	*p* < 0.01	−0.307	*p* < 0.066	−0.025	*p* = 0.674	0.99
Time and financial dimension (W3)	4.404	*p* < 0.01	−0.126	*p* = 0.358	0.015	*p* = 0.911	0.72
Negative attitude dimension (W4)	2.145	*p* < 0.01	0.006	*p* = 0.885	0.156	*p* = 0.179	0.92

## Data Availability

Not applicable.
